# Potential Treatments for COVID-19 Related Cytokine Storm - Beyond Corticosteroids

**DOI:** 10.3389/fimmu.2020.01445

**Published:** 2020-06-16

**Authors:** Yi Miao, Lei Fan, Jian-Yong Li

**Affiliations:** ^1^Department of Hematology, The First Affiliated Hospital of Nanjing Medical University, Jiangsu Province Hospital, Nanjing, China; ^2^Key Laboratory of Hematology of Nanjing Medical University, Nanjing, China

**Keywords:** COVID-19, hemophagocytic lymphohistiocytosis, IL-6, ruxolitinib, tocilizumab

A novel virus, SARS-CoV-2, emerged in Wuhan in December 2019 and rapidly spread to other cities in China and other countries. Several studies have summarized the clinical characteristics and laboratory findings of patients with Corona Virus Disease 2019 (COVID-19) (1, 2). According to these studies, in addition to lung injury, damages involving other organs, which include liver, kidney, heart, and hemopoietic system, were also observed in some patients, suggesting the presence of systemic inflammation, and from the work by Huang et al. (1), we noted that elevation of various proinflammatory cytokines was present in patients infected with SARS-CoV-2, suggesting the possible existence of cytokine storm in a proportion of patients. Further, patients that require intensive care unit (ICU) admission showed higher concentrations of certain cytokines compared with those not requiring ICU admission, indicating that the levels of proinflammatory cytokines were associated with disease severity. Further studies confirmed that levels of cytokines including interleukin (IL)-6 and IL-8 correlated with the disease severity of COVID-19 (3, 4). This phenomenon is not restricted to COVID-19, in the previous studies regarding the Middle East respiratory syndrome (MERS) and severe acute respiratory syndrome (SARS), higher levels of certain cytokines were associated with increased mortality (5, 6). For instance, high IL-6 concentration predicted mortality in patients with MERS (5). In patients infected with pathogenic human coronaviruses, cytokine storm contributes to acute lung injury and acute respiratory distress syndrome (ARDS) (7). Therefore, controlling the cytokine storm might be a strategy for treating patients with COVID-19, especially for those severe cases.

## Potential Treatments

Corticosteroids could be used to suppress the cytokine storm and have been used in some patients (1). However, based on the evidence from patients with MERS and ARDS, the use of corticosteroids did not provide a survival benefit but rather delayed the clearance of the virus, therefore, the systemic use of corticosteroids is not recommended by the WHO guidance (1). As a result, alternatives for dampening the overwhelming cytokine release are required.

As we know, the cytokine storm also occurs in other settings. In patients with leukemia or lymphoma who receive chimeric antigen receptor (CAR) T cells therapy, cytokine release syndrome (CRS) occurs during and after the infusion of CAR T cells (8). In patients receiving CAR T cells therapy, those with CRS had elevated concentrations of interferon γ, tumor necrosis factor α, interleukin (IL)-1B, IL-2, IL-6, IL-7, IL-8, IL-10, IL-12, granulocyte macrophage colony stimulating factor (GM-CSF), and macrophage inflammatory protein (MIP)-1. The cytokine profile in CRS related to CAR T cells infusion is similar to that in cases of SARS-CoV-2 infection. The anti-IL-6 receptor antibody tocilizumab is effective in controlling CAR T cells infusion related CRS (response rate: 53–69%) (9). The above evidence provides us with a rationale for using tocilizumab to manage the cytokine storm in patients with SARS-CoV-2 infection. Another rationale for using tocilizumab to treat COVID-19 is that IL-6 does not enhance the antiviral immunity but decreases the antiviral immunity in patients with COVID-19. Diao et al. found that serum IL-6 was negatively correlated with T cell numbers (10). Mazzoni et al. found that the elevation of IL-6 serum levels was associated with the impairment of cytotoxic activity in patients with COVID-19, and the use of tocilizumab restored the cytotoxic potential of NK cells (11). Some studies involving off-label use of tocilizumab have shown the potential efficacy of this drug in the treatment of COVID-19 (12–15).

Another potential drug that could be considered to treat cytokine storm is etoposide, which is used to deplete monocytes and suppress cytokine release in hemophagocytic lymphohistiocytosis (HLH) (16). It needs to be mentioned that, in SARS-CoV-infected mice, inflammatory monocyte-macrophage responses were involved in causing lethal pneumonia, suggesting the importance of suppressing monocyte-macrophage system in treating severe pneumonia related to SARS-CoV (17). The hyperactivation of monocytes/macrophages has been described in patients with COVID-19. Single-cell analysis of bronchoalveolar fluid revealed significantly increased proportions of mononuclear phagocytes in patients with COVID-19, especially those with severe disease. In patients with severe disease, these mononuclear phagocytes showed a predominance of inflammatory monocyte-derived macrophages (18). These macrophages could not only contribute to acute inflammation but also promote fibrosis generation. Additionally, a significant increase of CD14^+^CD16^+^ monocytes was also detected in patients with severe COVID-19 (19). These CD14^+^CD16^+^ monocytes expressed IL-6 and caused the acceleration of the inflammation. Therefore, etoposide could be used to inhibit the hyperactivation of monocytes/macrophages to suppress the overwhelming inflammation and ameliorate the pulmonary fibrosis. Other potential drugs for treating cytokine storm include the JAK1/2 inhibitor ruxolitinib, which is effective in inhibiting monocyte activation and cytokine release in patients with HLH (20). A prospective randomized study has shown the promising efficacy of ruxolitinib in the treatment of severe COVID-19 (21). In this trial, the ruxolitinib group showed a significant decrease of levels of 7 cytokines compared to the control group, suggesting ruxolitinib suppress the cytokine storm in patients with severe COVID-19. Patients in the ruxolitinib group also had a faster chest CT improvement and a faster recovery from lymphopenia. Ruxolitinib was also well-tolerated in patients with severe COVID-19, indicating ruxolitinib could be safely used to treat patients with COVID-19 (21). Additionally, therapeutic plasma exchange can reduce the plasma cytokine concentrations rapidly, and has been successfully used to treat HLH and CRS related to CAR T cells infusion (22, 23), suggesting plasma exchange may be a reasonable option for severe patients with cytokine storm. In a preliminary study, therapeutic plasma exchange reduced the plasma IL-6 level and improved the oxygenation status in patients with severe COVID-19 who had ARDS (24).

## Conclusion

Although we admit that supportive care and antiviral therapy remain the mainstay for treating patients with COVID-19, we recommend that treatments for controlling cytokine storm including tocilizumab, etoposide, ruxolitinib, and plasma exchange should be considered in selected COVID-19 patients with cytokine storm. Some pilot studies have shown promising results. Some other treatments may also be effective in controlling the cytokine storm. More randomized clinical trials are needed to evaluate if these treatments could reduce the mortality of patients with COVID-19.

## Author Contributions

YM, LF, and J-YL drafted the manuscript. All authors contributed to the article and approved the submitted version.

## Conflict of Interest

The authors declare that the research was conducted in the absence of any commercial or financial relationships that could be construed as a potential conflict of interest.
